# Linear stability in networks of pulse-coupled neurons

**DOI:** 10.3389/fncom.2014.00008

**Published:** 2014-02-04

**Authors:** Simona Olmi, Alessandro Torcini, Antonio Politi

**Affiliations:** ^1^Consiglio Nazionale delle Ricerche, Istituto dei Sistemi ComplessiSesto Fiorentino, Italy; ^2^INFN—Sezione di Firenze and CSDCSesto Fiorentino, Italy; ^3^SUPA and Institute for Complex Systems and Mathematical Biology, King's College, University of AberdeenAberdeen, UK

**Keywords:** linear stability analysis, splay states, synchronization, neural networks, pulse coupled neurons, Floquet spectrum

## Abstract

In a first step toward the comprehension of neural activity, one should focus on the stability of the possible dynamical states. Even the characterization of an idealized regime, such as that of a perfectly periodic spiking activity, reveals unexpected difficulties. In this paper we discuss a general approach to linear stability of pulse-coupled neural networks for generic phase-response curves and post-synaptic response functions. In particular, we present: (1) a mean-field approach developed under the hypothesis of an infinite network and small synaptic conductances; (2) a “microscopic” approach which applies to finite but large networks. As a result, we find that there exist two classes of perturbations: those which are perfectly described by the mean-field approach and those which are subject to finite-size corrections, irrespective of the network size. The analysis of perfectly regular, asynchronous, states reveals that their stability depends crucially on the smoothness of both the phase-response curve and the transmitted post-synaptic pulse. Numerical simulations suggest that this scenario extends to systems that are not covered by the perturbative approach. Altogether, we have described a series of tools for the stability analysis of various dynamical regimes of generic pulse-coupled oscillators, going beyond those that are currently invoked in the literature.

## 1. Introduction

Networks of oscillators play an important role in both biological (neural systems, circadian rhythms, population dynamics) (Pikovsky et al., 2003) and physical contexts (power grids, Josephson junctions, cold atoms) (Hadley and Beasley, 1987; Filatrella et al., 2008; Javaloyes et al., 2008). It is therefore comprehensible that many studies have been and are still devoted to understanding their dynamical properties. Since the development of sufficiently powerful tools and the resulting discovery of general laws is an utterly difficult task, it is convenient to start from simple setups.

The first issue to consider is the model structure of the single oscillators. Since phases are typically more sensitive than amplitudes to mutual coupling, they are likely to provide the most relevant contribution to the collective evolution (Pikovsky et al., 2003). Accordingly, here we restrict our analysis to oscillators characterized by a single, phase-like, variable. This is typically done by reducing the neuronal dynamics to the evolution of the membrane potential and introducing the corresponding *velocity field* which describes the single-neuron activity. Equivalently, one can map the membrane potential onto a phase variable and simultaneously introduce a phase-response curve (PRC) [Upon changing variables, the velocity field can be made independent of the local variable (as intuitively expected for a true phase). When this is done, the phase dependence of the velocity field is moved to the coupling function, i.e., to the PRC] to take into account the dependence of the neuronal response on the current value of the membrane potential (i.e., the phase). In this paper we adopt the first point of view, with a few exceptions, when the second one is mathematically more convenient.

As for the coupling, two mechanisms are typically invoked in the literature, diffusive and pulse-mediated. While the former mechanism is pretty well understood [see e.g., the very many papers devoted to Kuramoto-like models (Acebrón et al., 2005)], the latter one, more appropriate in neural dynamics, involves a series of subtleties that have not yet been fully appreciated. This is why here we concentrate on pulse-coupled oscillators.

Finally, for what concerns the topology of the interactions, it is known that they can heavily influence the dynamics of the neural systems leading to the emergence of new collective phenomena even in weakly connected networks (Timme, 2006), or of various types of chaotic behavior, ranging from weak chaos for diluted systems (Popovych et al., 2005; Olmi et al., 2010) to extensive chaos in sparsely connected ones (Monteforte and Wolf, 2010; Luccioli et al., 2012). We will, however, limit our analysis to globally coupled identical oscillators, which provide a much simplified, but already challenging, test bed. The high symmetry of the corresponding evolution equations simplifies the identification of the stationary solutions and the analysis of their stability properties. The two most symmetric solutions are: (1) the fully synchronous state, where all oscillators follow exactly the same trajectory; (2) the splay state (also known as “ponies on a merry-go-round,” antiphase state or rotating waves) (Hadley and Beasley, 1987; Ashwin et al., 1990; Aronson et al., 1991), where the oscillators still follow the same periodic trajectory, but with different (evenly distributed) time shifts. The former solution is the simplest representative of the broad class of clustered states (Golomb and Rinzel, 1994), where several oscillators behave in the same way, while the latter is the prototype of asynchronous states, characterized by a smooth distribution of phases (Renart et al., 2010).

In spite of the many restrictions on the mathematical setup, the stability of the synchronous and splay states still depend significantly on additional features such as the synaptic response-function, the velocity field, and the presence of delay in the pulse transmission. As a result, one can encounter splay states that are either strongly stable along all directions, or that present many almost-marginal directions, or, finally, that are marginally stable along various directions (Nichols and Wiesenfield, 1992; Watanabe and Strogatz, 1994). Several analytic results have been obtained in specific cases, but a global picture is still missing: the goal of this paper is to recompose the puzzle, by exploring the role of the velocity field (or, equivalently, of the phase response curve) and of the shape of the transmitted post-synaptic potentials. Although we are neither going to discuss the role of delay nor that of the network topology, it is useful to recall the stability analysis of the synchronous state in the presence of delayed δ-pulses and for arbitrary topology, performed by Timme and Wolf in Timme and Wolf (2008). There, the authors show that even the complete knowledge of the spectrum of the linear operator does not suffice to address the stability of the synchronized state.

The stability analysis of the fully synchronous regime is far from being trivial even for a globally coupled network of oscillators with no delay in the pulse transmission: in fact, the pulse emission introduces a discontinuity which requires separating the evolution before and after such event. Moreover, when many neurons spike at the same time, the length of some interspike intervals is virtually zero but cannot be neglected in the mathematical analysis. In fact, the first study of this problem was restricted to excitatory coupling and δ-pulses (Mirollo and Strogatz, 1990). In that context, the stability of the synchronous state follows from the fact that when the phases of two oscillators are sufficiently close to one another, they are instantaneously reset to the same value (as a result of a non-physical lack of invertibility of the dynamics). The first, truly linear stability analyses have been performed later, first in the case of two oscillators (van Vreeswijk et al., 1994; Hansel et al., 1995) and then considering δ-pulses with continuous PRCs (Goel and Ermentrout, 2002). Here, we extend the analysis to generic pulse-shapes and discontinuous PRCs [such as for leaky integrate and fire (LIF) neurons].

As for the splay states, their stability can be assessed in two ways: (1) by assuming that the number of oscillators is infinite (i.e., taking the so called thermodynamic limit) and thereby studying the evolution of the distribution of the membrane potentials—this approach is somehow equivalent to dealing with (macroscopic) Liouville-type equations in statistical mechanics; (2) by dealing with the (microscopic) equations of motion for a large but finite number *N* of oscillators. As shown in some pioneering works (Kuramoto, 1991; Treves, 1993), the former approach corresponds to develop a mean field theory. The resulting equations have been first solved in Abbott and van Vreeswijk (1993) for pulses composed of two exponential functions, in the limit of a small effective coupling [A small effective coupling can arise also when PRC has a very weak dependence on the phase (see section 3)]. Here, following Abbott and van Vreeswijk (1993), we extend the analysis to generic pulse-shapes, finding that substantial differences exist among δ, exponential and the so-called α-pulses (see the next section for a proper definition).

Direct numerical studies of the linear stability of finite networks suggest that the eigenfunctions of the (Floquet) operator can be classified according to their wavelength ℓ (where ℓ refers to the neuronal phase—see section 4.1 for a precise definition). In finite systems, it is convenient to distinguish between long (LW) and short (SW) wavelengths. Upon considering that ℓ = *n/N* (1 ≤ *n* ≤ *N*), LW can be identified as those for which *n* « *N*, while SW correspond to larger *n* values. Numerical simulations suggest also that the time scale of a LW perturbation typically increases upon increasing its wavelength, starting from a few milliseconds (for small *n* values) up to much longer values (when *n* is on the order of the network size *N*) which depend on “details” such as the continuity of the velocity field, or the pulse shape. On the other hand, SW are characterized by a slow size-dependent dynamics.

For instance, in LIF neurons coupled via α-pulses, it has been found (Calamai et al., 2009) that the Floquet exponents of LW decrease as 1/ℓ^2^ (for large ℓ), while the time scale of the SW component is on the order of *N*^2^. In practice the LW spectral component as determined from the finite *N* analysis coincides with that one obtained with the mean field approach (i.e., taking first the thermodynamic limit). As for the SW component, it cannot be quantitatively determined by the mean-field approach, but it is nevertheless possible to infer the correct order of magnitude of this time scale. In fact, upon combining the 1/ℓ^2^ decay (predicted by the mean-field approach) with the observation that the minimal wavelength is 1/*N*, it naturally follows that the SW time scale is *N*^2^, as analytically proved in Olmi et al. (2012). Furthermore, it has been found that the two spectral components smoothly connect to each other and the predictions of the two theoretical approaches coincide in the crossover region.

It is therefore important to investigate whether the same agreement extends to more generic pulse shapes and velocity fields. The finite-*N* approach can, in principle, be generalized to arbitrary shapes, but the analytic calculations would be quite lengthy, due to the need of distinguishing between fast and slow scales and the need of accounting for higher order terms. For this reason, here we limit ourselves to give a positive answer to this question with the help of numerical studies.

The only, important, exception to this scenario is obtained for quasi δ-like pulses (Zillmer et al., 2007), i.e., for pulses whose width is smaller than the average time separation between any two consecutive spikes, in which case all the SW eigenvalues remain finite for increasing *N*.

In section 2 we introduce the model and derive the corresponding event-driven map, a necessary step before undertaking the analytic calculations. Section 3 is devoted to a perturbative stability analysis of the splay state in the infinite-size limit for generic velocity fields and pulse shapes. The following section 4 reports a discussion of the stability in finite networks. There we briefly recall the main results obtained in Olmi et al. (2012) for the splay state and we extensively discuss the method to quantify the stability of the fully synchronous regime. The following two sections are devoted to a numerical analysis of various setups. In section 5 we study splay states in finite networks for generic velocity fields and three different classes of of pulses, namely, with finite, vanishing (≈1/*N*), and zero width. In section 6 we study periodically forced networks. Such studies show that the scaling relations derived for the splay states apply also to such a microscopically quasi-periodic regime. A brief summary of the main results together with a recapitulation of the open problem is finally presented in section 7. In the first appendix we derive the Fourier components needed to assess the stability of a splay state for a generic PRC. In the second appendix the evaporation exponent is determined for the synchronous state in LIF neurons.

## 2. The model

The general setup considered in this paper is a network of *N* identical pulse-coupled neurons (rotators), whose evolution is described by the equation

(1) $${\dot{X}}^{j}=F(X^{j})+gE(t),\;j=1,\ldots ,N$$

where *X*^*j*^ represents the membrane potential, *g* is the coupling constant and the *mean field E*(*t*) denotes to the synaptic input, common to all neurons in the network. When *X*^*j*^ reaches the threshold value *X*^*j*^ = 1, it is reset to *X*^*j*^ = 0 and a spike contributes to the mean field *E* in a way that is described here below. The resetting procedure is an approximation of the discharge mechanism operating in real neurons. The function *F*(*X*) (the velocity field) is assumed to be everywhere positive, thus ensuring that the neuron is repetitively firing. For *F*_0_(*X*) = *a* − *X* the model reduces to the well-known case of LIF neurons.

The mean field *E*(*t*) arises from the linear superposition of the pulses emitted by the single neurons. In order to describe its time evolution, it is sufficient to introduce a suitable ordinary differential equation (ODE), such that its Green function reproduces the expected pulse shape,

(2) $$E^{\left(L\right)}=\sum_{i}^{L-1}a_{i}E^{\left(i\right)}+\frac{K}{N}\sum_{n|t_{n}<t}\delta (t-t_{n}),$$

where the superscript (*i*) denotes the *i*th time derivative, *L* the order of the differential equation and *K* = ∏_*i*_ α_*i*_, (−α_*i*_ being the poles of the differential equation), so as to ensure that the single pulses have unit area (for *N* = 1). The δ-functions appearing on the right hand side of Equation (2) correspond to the spikes emitted at times {*t*_*n*_}: each time a spike is emitted, the term *E*^(*L* − 1)^ has a finite jump of amplitude *K/N*. Therefore *L* controls the smoothness of the pulses: *L* − 1 is the order of the lowest derivative that is discontinuous. *L* = 0 corresponds to the extreme case of δ-pulses with no field dynamics; *L* = 1 corresponds to discontinuous exponential pulses; *L* = 2 (with α_1_ = α_2_) to the so-called α-pulses (*E*_*s*_(*t*) = α^2^*te*^−α*t*^). Since α-pulses will be often referred to, it is worth being a little more specific. In this case, Equation (2) reduces to

(3) $$\ddot{E}(t)+2\alpha \dot{E}(t)+\alpha^{2}E(t)=\frac{\alpha^{2}}{N}\sum_{n|t_{n}<t}\delta (t-t_{n}),$$

and it is convenient to transform this equation into a system of two ODEs, namely

(4) $$\dot{E}=P-\alpha E,\;\dot{P}+\alpha P=\frac{\alpha^{2}}{N}\sum_{n|t_{n}\;<\;t}\delta (t-t_{n}),$$

where we have introduced, for the sake of simplicity, the auxiliary variable *P* ≡ α*E* + *Ė*.

### 2.1. Event-driven map

By following Zillmer et al. (2006) and Calamai et al. (2009), it is convenient to pass from a continuous—to a discrete-time evolution rule, by deriving the event-driven map which connects the network configuration at consecutive spike times. For the sake of simplicity, in the following part of this section we refer to α-pulses, but there is no conceptual limitation in extending the approach to *L* > 2.

By integrating Equation (4), we obtain

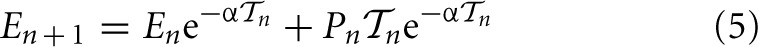

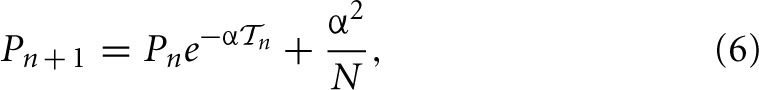





where we have taken into account the effect of the incoming pulse (see the term α^2^/*N* in the second equation) while 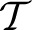
_*n*_ = *t*_*n* + 1_ − *t*_*n*_ is the interspike interval; *t*_*n*+1_ corresponds to the time when the neuron with the largest membrane potential reaches the threshold.

Since all neurons follow the same first-order differential equation (this is a mean-field model), the ordering of their membrane potentials is preserved [neurons “rotate” around the circle [0, 1] without overtaking each other (Jin, 2002)]. It is, therefore, convenient to order the potentials from the largest to the smallest one and to introduce a co-moving reference frame, i.e., to shift backward the label *j*, each time a neuron reaches the threshold. By formally integrating Equation (1),

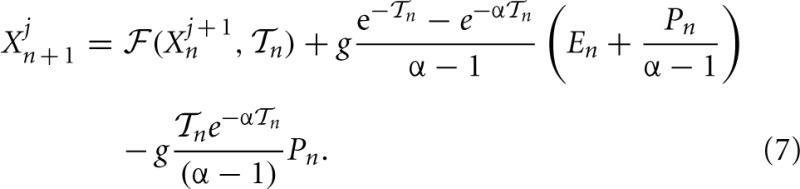



Moreover, since *X*^1^_*n*_ is always the largest potential, the interspike interval is defined by the threshold condition

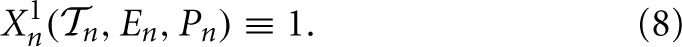



Altogether, the model now reads as a discrete-time map, involving *N* + 1 variables, *E*_*n*_, *P*_*n*_, and *X*^*j*^_*n*_ (1 ≤ *j* < *N*), since one degree of freedom has been eliminated as a result of having taken the Poincaré section (*X*^*N*^_*n*_ ≡ 0 due to the resetting mechanism). The advantage of the map description is that we do not have to deal any longer with δ-like discontinuities, or with formally infinite sequences of past events.

In this framework, the splay state is a fixed point of the event-driven map. Its coordinates can be determined in the following way. From Equation (5), one can express $\tilde{P}$ and *Ẽ* as a function of the yet unknown interspike interval 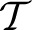,

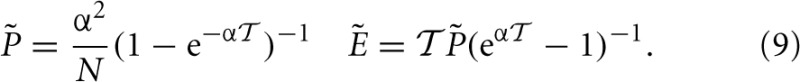



The value of the membrane potentials $\tilde{X}$^*k*^ are then obtained by iterating backward in *j* Equation (7) (the *n* dependence is dropped for the fixed point) starting from the initial condition $\tilde{X}$^*N*^ = 0. The interspike interval 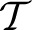 is finally obtained by imposing the condition $\tilde{X}$^0^ = 0. In practice the computational difficulty amounts to finding the zero of a one dimensional function and, even though 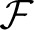
(*X*^*j* + 1^,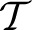) can, in most cases, be obtained only through numerical integration, the final error can be very well kept under control.

## 3. Theory (*N* = ∞)

The stability of a dynamical state can be assessed by either first taking the infinite-time limit and then the thermodynamic limit, or vice versa. In general it is not obvious whether the two methods yield the same result and this is particularly crucial for the splay state, as many eigenvalues tend to 0 for *N* → ∞. In this section we discuss the scenarios that have to be expected when the thermodynamic limit is taken first. We do that by following Abbott and van Vreeswijk (1993).

As a first step, it is convenient to introduce the phase-like variable

(10) $$y^{i}=\int_{0}^{X^{i}}\frac{dx}{G(x)},\;0\leq y^{i}\leq 1$$

where, for later convenience, we have defined *G*(*X*) ≡ *g* + *T*_0_ *F(X), T*_0_ = *N*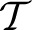 being the period of the splay state (i.e., the single-neuron interspike interval). The phase *y*^*i*^ evolves according to the equation

(11) $$\frac{dy^{i}}{dt}=\tilde{E}+\frac{g\varepsilon (t)}{G(X(y^{i}))}$$

where *Ẽ* = 1/*T*_0_ is the amplitude of the field in the splay state, ε(*t*) = *E*(*t*) − *Ẽ*. In the splay state, since ε = 0, *y*^*i*^ grows linearly in time, as indeed expected for a well-defined phase. In the thermodynamic limit, the evolution is ruled by the continuity equation

(12) $$\frac{\partial \rho }{\partial t}=-\frac{\partial J}{\partial y}$$

where ρ(*y, t*)*dy* is the fraction of neurons whose phase *y*^*i*^ lies in (*y, y* + *dy*) at time *t*, and

(13) $$J(y,t)=\left[\tilde{E}+\frac{g\varepsilon (t)}{G(X(y))}\right]\rho (y,t)$$

is the corresponding flux. As the resetting implies that the outgoing flux *J*(1, *t*) (which coincides with the firing rate) equals the incoming flux at the origin, the above equation has to be complemented with the boundary condition *J*(0, *t*) = *J*(1, *t*). Finally, in this macroscopic representation, the field equation writes

(14) $$\varepsilon^{\left(L\right)}=\sum_{i}^{L-1}a_{i}\varepsilon^{\left(i\right)}+K(J(1,t)-\tilde{E}),$$

while the splay state corresponds to the fixed point ρ = 1, ε = 0, *J* = *Ẽ*. The smoothness of the splay state justifies the use of a partial differential equation such as (Equation 12). Its stability can be studied by introducing the perturbation *j*(*y, t*)

(15) $$j(y,t)=J(y,t)-\tilde{E},$$

and linearizing the continuity equation,

(16) $$\frac{\partial j}{\partial t}=\frac{g}{G(X(y))}\frac{\partial \varepsilon }{\partial t}-\tilde{E}\frac{\partial j}{\partial y}.$$

while the field equation simplifies to

(17) $$\varepsilon^{\left(L\right)}=\sum_{i}^{L-1}a_{i}\varepsilon^{\left(i\right)}+Kj(1,t).$$

By now introducing the Ansatz

(18) $$j(y,t)=j_{f}(y){\text{e}}^{\lambda t}\;\varepsilon (y,t)=\varepsilon_{f}(y){\text{e}}^{\lambda t},$$

in Equations (16) and (17) and, thereby solving the resulting ODE, one can obtain an implicit expression for *j*_*f*_(*y*),

$$j_{f}(y)={\text{e}}^{-\lambda y/\bar{E}}\left[1+\frac{gK\lambda \;j_{f}(1)}{\tilde{E}\prod_{k=1}^{L}\left(\lambda +\alpha_{k}\right)}\int_{0}^{y}dz\frac{{\text{e}}^{\lambda z/\tilde{E}}}{G(X(z))}\right]\text{​},$$

where −α_*k*_ and *K* are defined as below Equation (2). By imposing the boundary condition for the flux, *j*_*f*_(1) = *j*_*f*_(0) = 1, one finally obtains the eigenvalue equation (Abbott and van Vreeswijk, 1993),

(19) $$\left({\text{e}}^{\lambda /\tilde{E}}-1\right)\prod_{k=1}^{L}\left(\lambda +\alpha_{k}\right)=\frac{gK\lambda }{\tilde{E}}\int_{0}^{1}dy\frac{{\text{e}}^{\lambda y/\tilde{E}}}{G(X(y))}.$$

In the case of a constant *G*(*X*(*y*)) = σ, *L* eigenvalues correspond to the zeroes of the following polynomial equation

(20) $$\prod_{k=1}^{L}\left(\lambda +\alpha_{k}\right)=\frac{gK}{\sigma }.$$

For *g* = 0 such solutions are the poles which define the field dynamics, while for *g* = σ, λ = 0 is a solution: this corresponds to the maximal value of the (positive) coupling strength beyond which the model does no longer support stationary states, as the feedback induces an unbounded growth of the spiking rate. Besides such *L* solution, the spectrum is composed of an infinite set of purely imaginary eigenvalues,

(21) $$\lambda =2\pi \text{in}\tilde{E}=\frac{2\pi \text{in}}{T_{0}}\;n\neq 0.$$

The existence of such marginally stable directions reflects the fact that all *y*^*i*^ phases experience the same velocity field, independently of their current value (see Equation 11), so that no effective interaction is present among the oscillators. In the limit of small variations of *G*(*X*(*y*)), one can develop a perturbative approach. Here below, we proceed under the more restrictive assumption that the coupling constant *g* is itself small: we have checked that this restriction does not change the substance of our conclusions, while requiring a simpler algebra.

A small *g* value implies that λ is close to 2πin*Ẽ* and thereby expand the exponential in Equation (19). Up to first order, we find

(22) $$\lambda_{n}=2\pi \text{in}\tilde{E}\left[1+\frac{gK(A_{n}+iB_{n})}{\prod_{k=1}^{L}\left(2\pi \text{in}\tilde{E}+\alpha_{k}\right)}\right]$$

where

(23) $$(A_{n}+iB_{n})=\int_{0}^{1}dy\frac{{\text{e}}^{i2\pi ny}}{G(X(y))}$$

are the Fourier components of the phase-response curve 1/*G(X(y))*.

In order to estimate the leading terms of the real part of λ_*n*_ in the large *n* limit, let us rewrite Equation (22) as

(24) $$\lambda_{n}=i\gamma_{n}+gK\gamma_{n}\frac{-B_{n}+iA_{n}}{\prod_{k=1}^{L}\left(\alpha_{k}^{2}+\gamma_{n}^{2}\right)}\prod_{k=1}^{L}\left(\alpha_{k}-i\gamma_{n}\right)$$

where γ_*n*_ = 2π*nẼ* = (2π*n*)/*T*_0_. Since γ_*n*_ is proportional to *n*, the leading terms in the product at numerator of Equation (24) are

(25) $$\prod_{k=1}^{L}\left(\alpha_{k}-i\gamma_{n}\right)\sim {\left(-i\right)}^{L}\gamma_{n}^{L}+S{\left(-i\right)}^{L-1}\gamma_{n}^{L-1},$$

where $S=\sum_{k\;=\;1}^{L}\alpha_{k}$ while the leading term in the product at denominator in Equation (24) is γ^2*L*^_*n*_. Accordingly, the main contribution to the real part of the eigenvalues is, in the case of even *L*,

(26) $$Re\{\lambda_{n}\}\sim gK{\left(-1\right)}^{L/2}\left[\frac{SA_{n}}{\gamma_{n}^{L}}-\frac{B_{n}}{\gamma_{n}^{L-1}}\right]$$

and, for odd *L*,

(27) $$Re\{\lambda_{n}\}\sim gK{\left(-1\right)}^{(L+3)/2}\left[\frac{A_{n}}{\gamma_{n}^{L-1}}+\frac{SB_{n}}{\gamma_{n}^{L}}\right]\text{​}.$$

An exact expression for the Fourier components *A*_*n*_ and *B*_*n*_ appearing in Equation (23) can be derived in the large *n* limit. In particular, the integral over the interval [0, 1] appearing in Equation (23) can be rewritten as a sum of integrals, each performed on a sub-interval of vanishingly small length 1/*n*. Furthermore, since the phase-response 1/*G* has a limited variation within each sub-interval, it can be replaced by its polynomial expansion up to second order. Finally, as shown in Appendix A, the following expression are obtained at the leading order in 1/*n* for a discontinuous *F*(*X*)

(28) $$A_{n}≃\frac{-T_{0}}{4\pi^{2}n^{2}}\left[\frac{F^{\prime }(1)}{G{\left(1\right)}^{2}}-\frac{F^{\prime }(0)}{G{\left(0\right)}^{2}}\right]\text{​},$$

(29) $$B_{n}≃\frac{T_{0}}{2\pi n}\left[\frac{F(1)-F(0)}{G(1)G(0)}\right]\text{​}.$$

Therefore, for even *L*, the leading term for *n* → ∞ is

(30) $$Re\{\lambda_{n}\}=\frac{g{\text{KT}}_{0}^{L}{\left(-1\right)}^{L/2}\left(F(0)-F(1)\right)}{{\left(2\pi n\right)}^{L}G(1)G(0)}.$$

For even *L*, the stability of the short-wavelength modes (large *n*) is controlled by the sign of (*F*(0) − *F*(1)): for even (odd) *L*/2 and excitatory coupling, i.e., *g* > 0, the splay state is stable whenever *F*(1) > *F*(0) (*F*(1) < *F*(0)). Obviously the stability is reversed for inhibitory coupling.

Notice that for *L* = 0, i.e., δ-spikes, the eigenvalues do not decrease with *n*, as previously observed in Zillmer et al. (2007). This is the only case where all modes exhibit a finite stability even in the thermodynamic limit.

For odd *L*, the real part of the eigenvalues is

(31) $$Re\{\lambda_{n}\}=\frac{g{\text{KT}}_{0}^{L}{\left(-1\right)}^{(L+1)/2}}{{\left(2\pi n\right)}^{\left(L+1\right)}}\times \left\{\frac{F^{\prime }(1)}{G{\left(1\right)}^{2}}-\frac{F^{\prime }(0)}{G{\left(0\right)}^{2}}-ST_{0}\frac{F(1)-F(0)}{G(1)G(0)}\right\}\text{​},$$

in this case the value of *F*(*X*) and of its derivative *F*′(*X*) at the extrema mix up in a non-trivial way.

Finally, as for the scaling behavior of the leading terms we observe that

(32) $$Re\{\lambda_{n}\}\sim n^{-q},\;q=2\left\lfloor \frac{L+1}{2}\right\rfloor$$

where ⌊·⌋ stays for the integer part of the number. Therefore the scaling of the short-wavelength modes for discontinuous *F*(*X*) is dictated by the post-synaptic pulse profile.

For a continuous but non-differentiable *F*(*X*), (i.e., *F*′(1) ≠ *F*′(0)), if *L* is even, it is necessary to go two orders beyond in the estimate of the Fourier coefficients (see Appendix A). As a result, the eigenvalues scale as

(33) $$Re\{\lambda_{n}\}\propto n^{-(L+2)}.$$

For odd *L*, it is instead sufficient to assume *F*(0) = *F*(1) in Equation (31).

Altogether, we have seen that the non-smoothness of both the post-synaptic pulse and of the velocity field (or, equivalently, of the phase response curve) play a crucial role in determining the degree of stability of the splay state. The smoother are such functions and the slower short-wavelength perturbations decay, although the changes occur in steps which depend on the parity of the order of the discontinuity (at least for the pulse structure). Moreover, the overall stability of the spectral components depends in a complicate way on the sign of the discontinuity itself.

## 4. Theory (finite *N*)

### 4.1. The splay state

The stability for finite *N* can be investigated by linearizing Equations (5–7). A thorough analysis has been developed in Olmi et al. (2012); here we limit ourselves to review the key ideas as a guide for the numerical analysis.

We start by introducing the vector *W* = ({*x*^*j*^}, ϵ, *p*) (*j* = 1, *N* − 1), whose components represent the infinitesimal perturbations of the solution {*X*^*j*^}, *E, P*. The Floquet spectrum can be determined by constructing the matrix **A** which maps the initial vector *W*(0) onto *W*(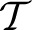),

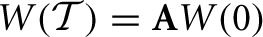



where 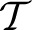 corresponds to the time separation between two consecutive spikes. This is done in two steps, the first of which corresponds to evolving the components of a Cartesian basis according to the equations obtained from the linearization of Equations (1, 4) (in the comoving reference frame),

(35) $$\begin{array}{l} {\dot{x}}^{j}=\frac{dF}{dx_{j+1}}x^{j+1}+g\epsilon ,\;j=2,\ldots ,N\;{\dot{x}}^{N}\equiv 0\\ \;\dot{\epsilon }=p-\alpha \epsilon ,\;\dot{p}=-\alpha p. \end{array}$$

The second step consists in accounting for the spike emission, which amounts to add the vector

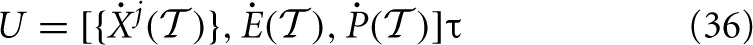



where τ is obtained from the linearization of the threshold condition (8),

(37) $$\tau =-\left(\frac{\partial X^{1}}{\partial E}\epsilon +\frac{\partial X^{1}}{\partial P}p\right)\frac{1}{{\dot{X}}^{1}}$$

The diagonalization of the resulting matrix **A**, gives *N* + 1 Floquet eigenvalues μ_*k*_, which we express as

(38) $$\mu_{k}=e^{i\phi_{k}}e^{T_{0}(\lambda_{k}+i\omega_{k})/N},$$

where $\phi_{k}=\frac{2\pi k}{N}$, *k* = 1, …, *N* − 1, and ϕ_*N*_ = ϕ_*N*−1_ = 0, while λ_*k*_ and ω_*k*_ are the real and imaginary parts of the Floquet exponents. The variable ϕ_*k*_ plays the role of the wavenumber *k* in the linear stability analysis of spatially extended systems.

Previous studies (Olmi et al., 2012) have shown that the spectrum can be decomposed into two components: (1) *k* ~ 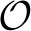
(1); (2) *k/N* ~ 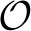
(1). The former one is the LW component and can be directly obtained in the thermodynamic limit (see the previous section). For *L* = 2 and α_1_ = α_2_ (i.e., for α pulses), it has been found that the results reported in Abbott and van Vreeswijk (1993) match does obtained for 1« *k* « *N* in Olmi et al. (2012). The latter one corresponds to the SW component: it depends on the system size and cannot, indeed, be derived from the mean field approach discussed in the previous section. In the next section, we illustrate some examples that go beyond the analytic studies carried out in Olmi et al. (2012).

### 4.2. The synchronized state

In this section we address the problem of measuring the stability of the fully synchronized state for a generic oscillator dynamics *F*(*x*). The task is non-trivial, because of the resetting mechanism, which acts simultaneously on all neurons. On the one side, we extend the results obtained in Goel and Ermentrout (2002) which are restricted to a continuous PRC, on the other side we extend the results of Mirollo and Strogatz (1990) which refer to excitatory coupling and δ pulses. In order to make the analysis easier to understand we start considering α-pulses. Other cases are discussed afterward.

The starting point amounts to writing the event driven map in a comoving frame,

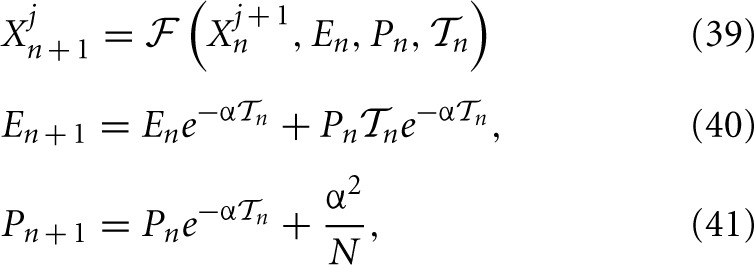



where the function 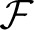 is obtained by formally integrating the equations of motion over the time interval 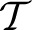
_*n*_. Notice that the field dynamics has been, instead, explicitly obtained from the exact integration of the equations of motion [compare with Equations (3, 4)]. The interspike time interval 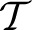
_*n*_ is finally determined by solving the implicit equation

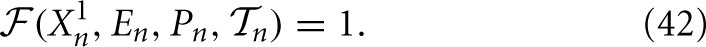



In order to determine the stability of the synchronized state, it is necessary to assume that the neurons have an infinitesimally different membrane potentials, even though they coincide with one another. As a result, the full period must be broken into *N* steps. In the first one, of length *T*, all neurons start in *X* = 0 and arrive at 1, but only the “first” reaches the threshold; in the following *N* − 1 steps, of 0-length, one neuron after the other passes the threshold and it is accordingly reset in 0.

With this scheme in mind we proceed to linearize the equations, writing the evolution equations for the infinitesimal perturbations *x*^*j*^_*n*_, ϵ_*n*_, *p*_*n*_, and τ_*n*_ around the synchronous solution. From Equations (39–41) we obtain,

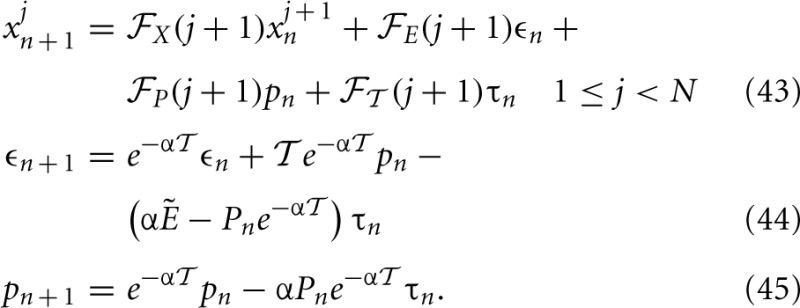



with the boundary condition *x*^*N*^_*n* + 1_ = 0 (due to the reset mechanism) and where the subscripts *X, E, P*, and 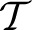 denote a partial derivative with respect to the given variable. Moreover, the dependence on *j* + 1 is a shorthand notation to remind that the various derivatives depend on the membrane potential of the (*j* + 1)st neuron. Finally, we have left the *n*-dependence in the variable *P* as it changes (in α^2^/*N* steps, when the neurons progressively cross the threshold), while *Ẽ* refers to the field amplitude, which, instead, stays constant.

The above equations must be complemented by the condition

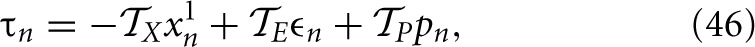



where 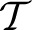
_*Z*_ = 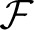
_*Z*_(1)/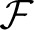
_
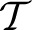
_(1) (*Z* = *X, E, P*). Equation (46) is obtained by differentiating Equation (42) which defines the period of the splay state.

We now proceed to build the Jacobian for each of the *N* steps, starting from the first one. In order not to overload the notations, from now on, the time index *n* corresponds to the step of the procedure. It is convenient to order all the variables, starting from *x*^*j*^ (*j* = 1, *N* − 1), and then including ϵ and *p*, into a single vector, so that the evolution is described by an (*N* + 1) × (*N* + 1) matrix with the following structure,

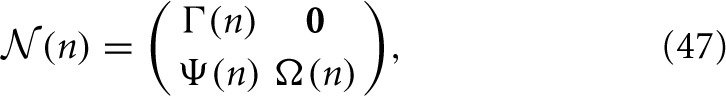



where **0** is an (*N* − 1) × 2 null matrix; Γ(*n*) is a quadratic (*N* − 1) × (*N* − 1) matrix, whose only non-zero elements are those in the first column and along the supradiagonal; Ψ(*n*) is a 2 × (*N* − 1) matrix whose elements are all zero except for the first column; finally Ω(*n*) is a 2 × 2 matrix.

Since in the first step all neurons start from the same position *X* = 0, one can drop the *j* dependence in 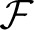. With the help of Equations (46, 43)

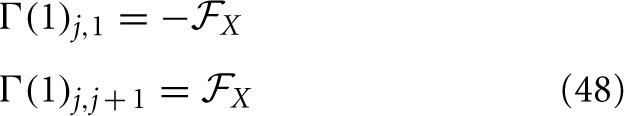



Moreover, with the help of Equations (44–46)

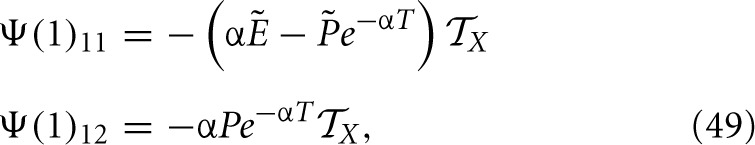



where we have also made use that *P*_1_ = $\tilde{P}$. Finally,

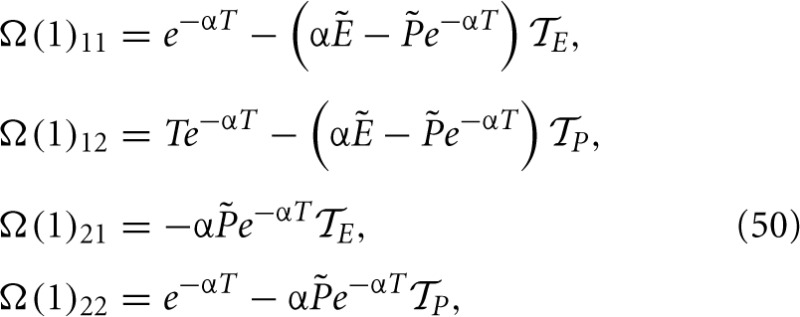



In the next steps, 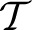
_*n*_ vanishes, so that 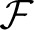
_*E*_ = 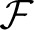
_*P*_ = 0, while 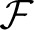
_*X*_ = 1 and 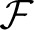
_
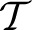
_(1) = *F*(1) + *gẼ*: = *V*^1^. Moreover, 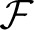
_
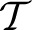
_(*j*) depends on whether the *j*th neuron has passed the threshold or not. In the former case 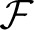
_
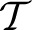
_(*j* + 1) = *F*(0) + *gẼ*: = *V*_0_, otherwise 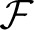
_
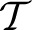
_(*j* + 1) = *V*^1^. As a result,

(51) $$\begin{array}{l} \Gamma {\left(n\right)}_{j,1}=-V^{j}/V^{1}\\ \Gamma {\left(n\right)}_{j,j+1}=1 \end{array}$$

where *V*^*j*^ = *V*^0^ if *j* < *n* and *V*^*j*^ = *V*^1^, otherwise. At the same time, from the equations for the field variables, we find that

(52) $$\begin{array}{l} \Psi {\left(n\right)}_{11}=\frac{\alpha \tilde{E}-(\tilde{P}+(n-1)\frac{\alpha^{2}}{N})}{V^{1}}\\ \Psi {\left(n\right)}_{12}=\frac{\alpha (\tilde{P}+(n-1)\frac{\alpha^{2}}{N})}{V^{1}}, \end{array}$$

while Ω(*n*) reduces to the identity matrix.

From the multiplication of all matrices, we find that the structure is preserved, namely

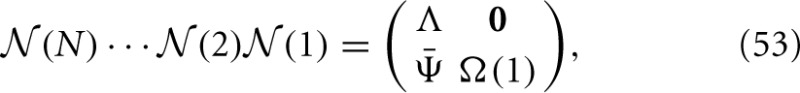



where Ψ(*n*) is a 2 × (*N* − 1) matrix, whose elements are all zero except for those of the first column, namely

$$\begin{array}{l} {\bar{\Psi }}_{11}=\Psi {\left(1\right)}_{11}+\Psi {\left(n\right)}_{11}\\ {\bar{\Psi }}_{12}=\Psi {\left(1\right)}_{12}+\Psi {\left(n\right)}_{12} \end{array}$$

Furthermore, Λ is a diagonal matrix, with

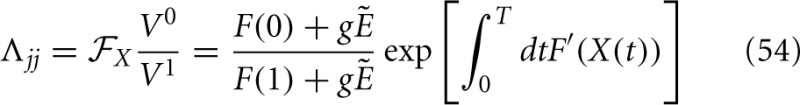



Therefore, it is evident that the stability of the orbit is measured by the diagonal elements Λ_*jj*_ together with the eigenvalues of Ω which are associated to the pulse structure. In practice, 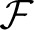
_*X*_ corresponds to the expansion rate from *X* = 0 to *X* = 1 under the action of the mean field *E* and we recover a standard result in globally coupled identical oscillators: the spectrum is degenerate, all eigenvalues being equal and independent of the network size. The result is, however, not obvious in this context, due to the care that is needed in taking into account the various discontinuities. We have separately verified that the same conclusion holds for exponential spikes.

The stability of the synchronized state can be also addressed by determining the evaporation exponent Λ_*e*_ (van Vreeswijk, 1996; Pikovsky et al., 2001), which measures the stability of a probe neuron subject to the mean field generated by the synchronous neurons with no feedback toward them. By implementing this approach for a negative perturbation, van Vreeswijk found that Λ_*e*_ is equal to Λ_*jj*_ (for α-functions). By further assuming that *F*′ < 0, he was able to prove that the synchronized state is stable for inhibitory coupling and sufficiently small α-values. The situation is more delicate for exponential pulse-shapes. As shown in di Volo et al. (2013), Λ_*e*_ > 0 (Λ_*e*_ < 0) depending whether the perturbation is positive (negative). In this case, the Floquet exponent reported in Equation (54) coincides with the evaporation exponent estimated for negative perturbations. In Appendix B we show that the difference between the left and right stability is to be attributed to the discontinuous shape of the pulse: no anomaly is expected for α pulses.

## 5. Numerical analysis

The theoretical approaches discussed in the previous sections allow determining: (1) the SW components of the Floquet spectrum for discontinuous velocity fields; (2) the leading LW exponents directly in the thermodynamic limit for generic velocity fields and pulse shapes, in the weak coupling limit. It would be possible to extend the finite *N* results to other setups, but we do not think that the effort is worth, given the huge amount of technicalities. We thus prefer to illustrate the expected behavior with the help of some simulations which, incidentally, cover a wider range than possibly accessible to the analytics.

More precisely, in this and the following section we study the models listed in Table 1 in a standard set up (splay states) and under the effect of periodic external perturbations.

**Table 1:** **In the first column is reported the list of the velocity fields *F*(*X*) analyzed in the paper. All the considered fields are everywhere positive within the definition interval *X* ∈ [0, 1], thus ensuring that the neuron is supra-threshold. The second column refers to the continuity properties of the fields within the interval [0, 1]**.

**Velocity field**	**Continuity properties**
*F*_0_(*X*) = *a − X*	Discontinuous
*F*_1_(*X*) = *a−X*(*X* − 0.7)	Discontinuous
*F*_2_(*X*) = *a* − 0.25sin(π*X*)	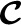 ^(0)^
*F*_3_(*X*) = *a* + *X*(*X* − 1)	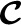 ^(0)^
*F*_4_(*X*) = *a* − 0.25 sin(π*X*)cos^2^(π*X*)	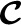 ^(0)^
*F*_5_(*X*) = *a* − 0.25 sin(2π*X*)cos^2^(2π*X*)	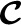 ^(∞)^
*F*_6_(*X*) = *a* − 0.25 sin(2π*X*)*e*^cos(2π*X*)^	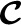 ^(∞)^
*F*_7_(*X*) = *a* − 1 + *e*^2sin(2π*X*)^	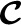 ^(∞)^

*The function is labeled as discontinuous if F(0)≠ F(1); it is* 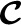
*^(0)^ if F(0) = F(1) but F'(0)≠ F'(1) and* 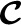
*^(1)^ if F(0) = F(1) and F'(0) = F'(1). F(X) is* 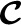
*^(∞)^ if it is infinitely differentiable and each derivative is continuous at the extrema of the definition interval*.

### 5.1. Finite pulse width

Here, we discuss the stability of the splay state for different degrees of smoothness of the velocity field at the borders of the unit interval for post-synaptic pulses of α-function type.

**Figure 1:**
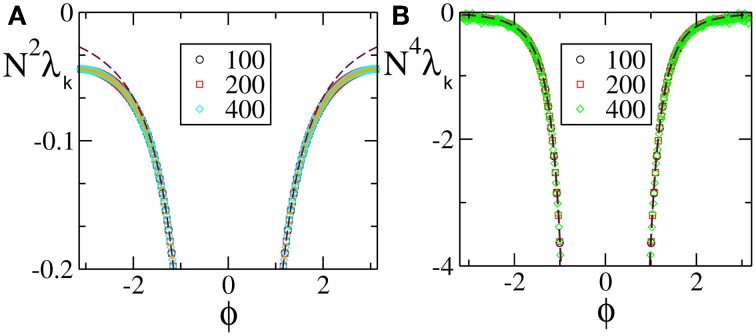
**Floquet spectra for α-pulses for (A) a discontinuous field *F*_1_(*X*) and (B) a continuous field *F*_4_(*X*)**. The orange dotted line in **(A)** represents the theoretical curve estimated by using Equation (7) in Olmi et al. (2012), while the dashed maroon curve represents the theoretical curve estimated by using Equation (30) in section 3. In **(B)** the dashed maroon curve is calculated by using Equation (A10). All data refer to *a* = 1.3 and α = 3.

We start from discontinuous velocity fields. They have been the subject of an analytic study which proved that the SW component scales as 1/*N*^2^ (Olmi et al., 2012). The data reported in Figure 1A for *F*_1_(*X*) confirms the expected scaling: the agreement with the theoretical curve derived in Olmi et al. (2012) is impressive over the entire spectral range, while the mean field Equation (30) gives a very good estimation of the spectrum except for the shortest wavelengths, where it overestimates the numerical data. The mean field approximation turns out to be more accurate for continuous velocity fields (with a discontinuity of the first derivative at the borders of the definition interval). Indeed the agreement between the theoretical expression Equation (A10) and the numerical data is very good for the entire range [see Figure 1B which refers to *F*_4_(*X*)].

**Figure 2:**
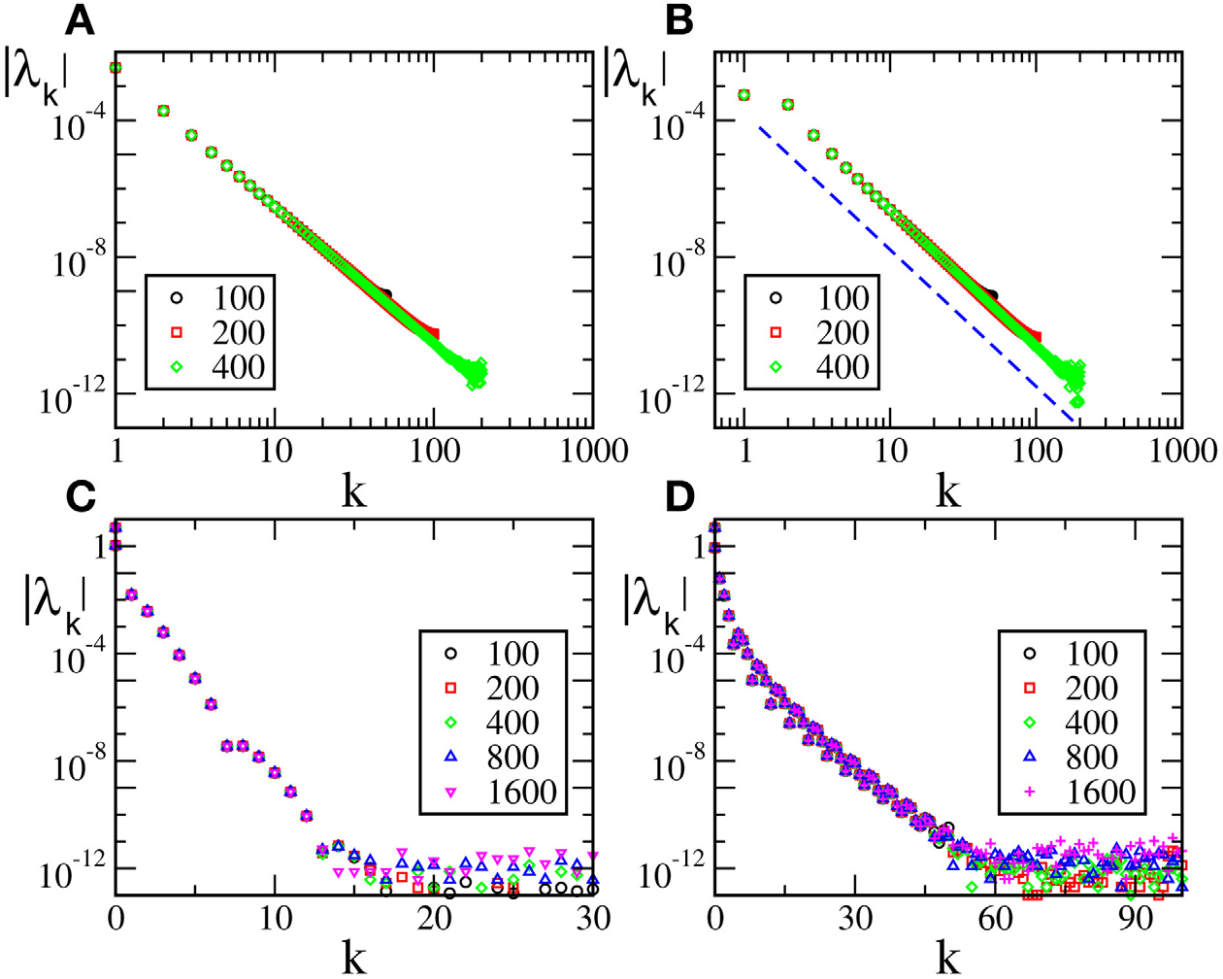
**Floquet spectra for α-pulses for two continuous sinusoidal fields, namely *F*_2_(*X*) (A) and *F*_4_(*X*) (B); and two analytic fields, namely *F*_6_(*X*) (C) and *F*_7_(*X*) (D)**. The dashed blue line in **(B)** indicates a scaling 1/*k*^4^. All data refer to *a* = 1.3 and α = 3.

The numerical Floquet spectra for fields that are 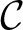
^(0)^, but not 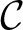
^(1)^ (*F*(0) = *F*(1), *F*′(0)≠ *F*′(1)), are reported in Figure 2 [the curves in panels (**A**, **B**) refer to *F*_2_(*X*) and *F*_4_, respectively]. For these velocity fields, we have also verified that the spectra scale as 1/*N*^4^, confirming the observation reported in Calamai et al. (2009) for a different velocity field with the same analytical properties. The data displayed in Figures 2A,B refer to the LW components: they indeed confirm to be independent of the system size and scale as 1/*k*^4^ (see the dashed line) as predicted by the perturbative theory discussed in section 3.

The spectra reported in the other two panels refer to analytic velocity fields: in all cases the initial part of the Floquet spectra is again independent of *N* and scales approximately exponentially with *k*, confirming that the scaling behavior of the exponents is related to the analyticity of the velocity field. The fluctuating background with approximate height 10^−12^ is just a consequence of the finite numerical accuracy. This is the reason why we did not dare to estimate the SW components that would be exceedingly small.

### 5.2. Vanishing pulse-width

Here, we analyze the intermediate case between finite pulse-width and δ-like impulses. Similarly to what done in Zillmer et al. (2007) for the LIF, we consider α pulses, where α = β*N*, with β independent of *N*.

**Figure 3:**
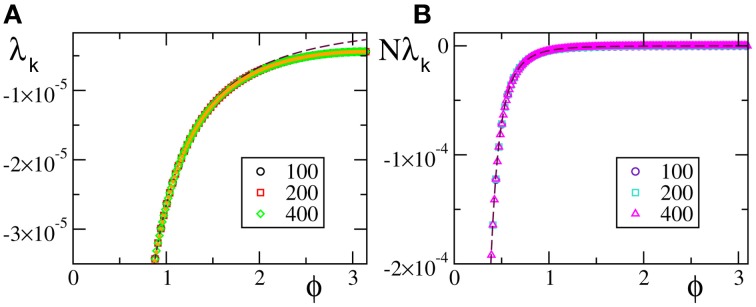
**Floquet spectra for β-pulses with a discontinuous field [*F*_1_(*X*)] (A) and a** 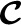
**^(0)^ field [*F*_2_(*X*)] (B)**. The orange dotted line in (**A**) represents the theoretical curve estimated by using Equation (7) in Olmi et al. (2012). The dashed line in (**A**) [resp. (**B**)] represents the theoretical curve computed by using Equation (30) [resp. Equation (A10)] for β-pulses. The data refer to *a* = 1.3 and β = 0.03.

In Figure 3A we report the spectra for a discontinuous velocity field, *F*_1_(*x*). In this case the Floquet spectra remain finite, so that the corresponding states remain robustly stable even in the thermodynamic limit. Also in this case the agreement with the theoretical expression reported in Equation (7) in Olmi et al. (2012) is extremely good, while Equation (30) overestimates the spectra for large phases. The field considered in panel (b) (*F*_2_(*X*)) is 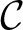
^(0)^ but not 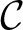
^(1)^. In this case, the Floquet spectra scale as 1/*N*: this scaling is predicted by the analysis reported in section 3 and the whole spectrum is very well reproduced by Equation (A10).

**Figure 4:**
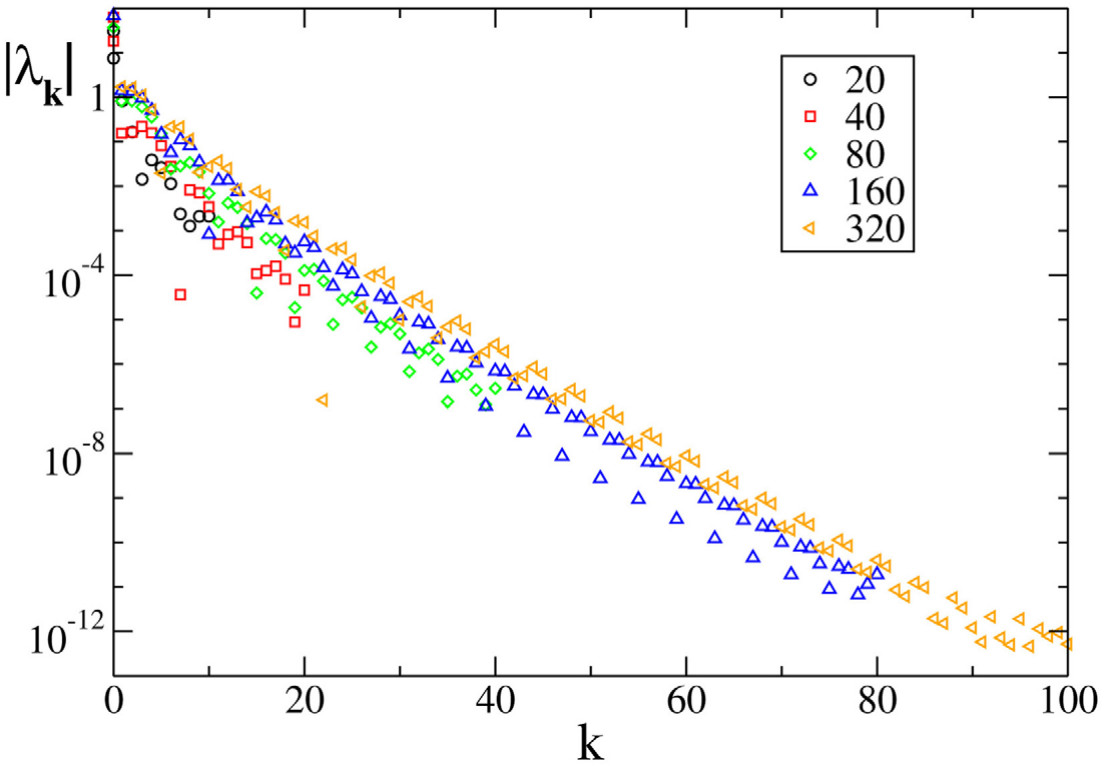
**Floquet spectra for β-pulses for the analytic field *F*_7_(*X*)**. The data refer to *a* = 1.3 and β = 0.03.

Last but not least, we have studied an analytic field, namely *F*_7_(*X*). In this case the Floquet spectra appear to scale exponentially to zero with the wavevector *k*, similarly to what observed for the finite pulse width, as shown in Figure 4.

### 5.3. δ pulses

Finally we considered the case of δ-pulses: whenever the potential *X*^*j*^ reaches the threshold value, it is reset to zero and a spike is sent to and *instantaneously* received by all neurons. We studied just two cases: (1) the analytic field *F*_7_(*X*); (2) a leaky integrate-and fire neuron model with *F*_0_(*X*). The results, obtained for inhibitory coupling [since the splay state is known to be stable only in such a case (van Vreeswijk, 1996; Zillmer et al., 2006)] are consistent with the expectation for the β model.

In particular we found, in the analytic case (1), that the Floquet spectra decay exponentially to zero. The exponential scaling is not altered if a phase shift ζ is introduced in the velocity field (i.e., for *F*(*X*) = *a* − 1 + *e*^2sin(2π*X* + ζ)^). In the case of the LIF model (*F*_0_), we already know that the Lyapunov spectrum tends, in the δ-pulse limit, to Zillmer et al. (2007)

(55) $$\underset{\beta \to \infty }{\lim}\lambda_{\pi }=-1+\frac{1}{T_{0}}\ln\left(\frac{a}{a-1}\right)\text{​}.$$

This result is confirmed by our simulations which also reveal that the splay state is stable even for small, excitatory coupling values, extending previous results limited to inhibitory coupling (Zillmer et al., 2006).

## 6. Periodic forcing

In this section we numerically investigate the scaling behavior of the Floquet spectrum in the presence of a periodic forcing, to test the validity of the previous analysis in a more general context. We have restricted our studies to splay-state-like regimes, where it is important to predict the behavior of the many almost marginally stable directions. Moreover, we have considered only the smooth α-pulses. In this case, the dynamical equations read

(56) $$\begin{array}{l} {\dot{X}}^{j}=F(X^{j})+gE+A\cos(\varphi ),\;j=1,\ldots ,N,\\ \;\dot{E}=P-\alpha E,\\ \;\dot{P}=-\alpha P,\\ \;\dot{\varphi }=\omega . \end{array}$$

They have been written in an autonomous form, since it is more convenient to perform the Poincaré section according to the spiking times, rather than introducing a stroboscopic map. The interspike interval is determined by the equation

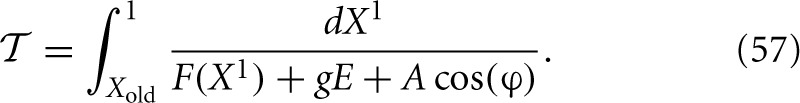



where *X*^1^ is the membrane potential of the first neuron (the closest to threshold), and *X*_old_ is its initial value.

We analyzed only those setups where the unperturbed splay state is stable. More precisely: the two discontinuous fields *F*_0_(*X*) and *F*_1_(*X*), the two 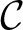
^(0)^ fields (*F*_2_(*X*) and *F*_3_(*X*)), and the analytic field *F*_7_(*X*). In all cases the external modulation induces a periodic modulation of the mean field *E* with a period *T*_*a*_ = 2π/ω equal to the period of the modulation. At the same time, we have verified that, although the forcing term has zero average (i.e., it does not change the average input current), the average interspike interval is slightly self-adjusted and, what is more important, there is no evidence of locking between the modulation and the frequency of the single neurons. In other words, the behavior is similar to the spontaneous partial synchronization observed in van Vreeswijk (1996) (where the modulation is self-generated).

Because of the unavoidable oscillations of the interspike intervals, it is necessary to identify the spike times with great care. In practice we integrate Equation (56) with a fixed time step Δ*t*, by employing a standard fourth-order Runge–Kutta integration scheme. At each time step we check if *X*^1^ > 1, in which case we go one step back and adopt the Hénon trick, which amounts to exchanging *t* and *X*^1^ in the role of independent variable (Henon, 1982).

The linear stability analysis can be performed by linearizing the system (56), to obtain

$$\begin{array}{l} \;{\dot{x}}^{j}=\frac{dF(X^{j})}{dX^{j}}x^{j}+g\epsilon -A\sin(\varphi )\delta \varphi ,\;j=1,\ldots ,N,\\ \;\dot{\epsilon }=p-\alpha \epsilon ,\\ \;\dot{p}=-\alpha p,\\ \;\delta \dot{\varphi }=0; \end{array}$$

and by thereby estimating the corresponding Lyapunov spectrum.

**Figure 5:**
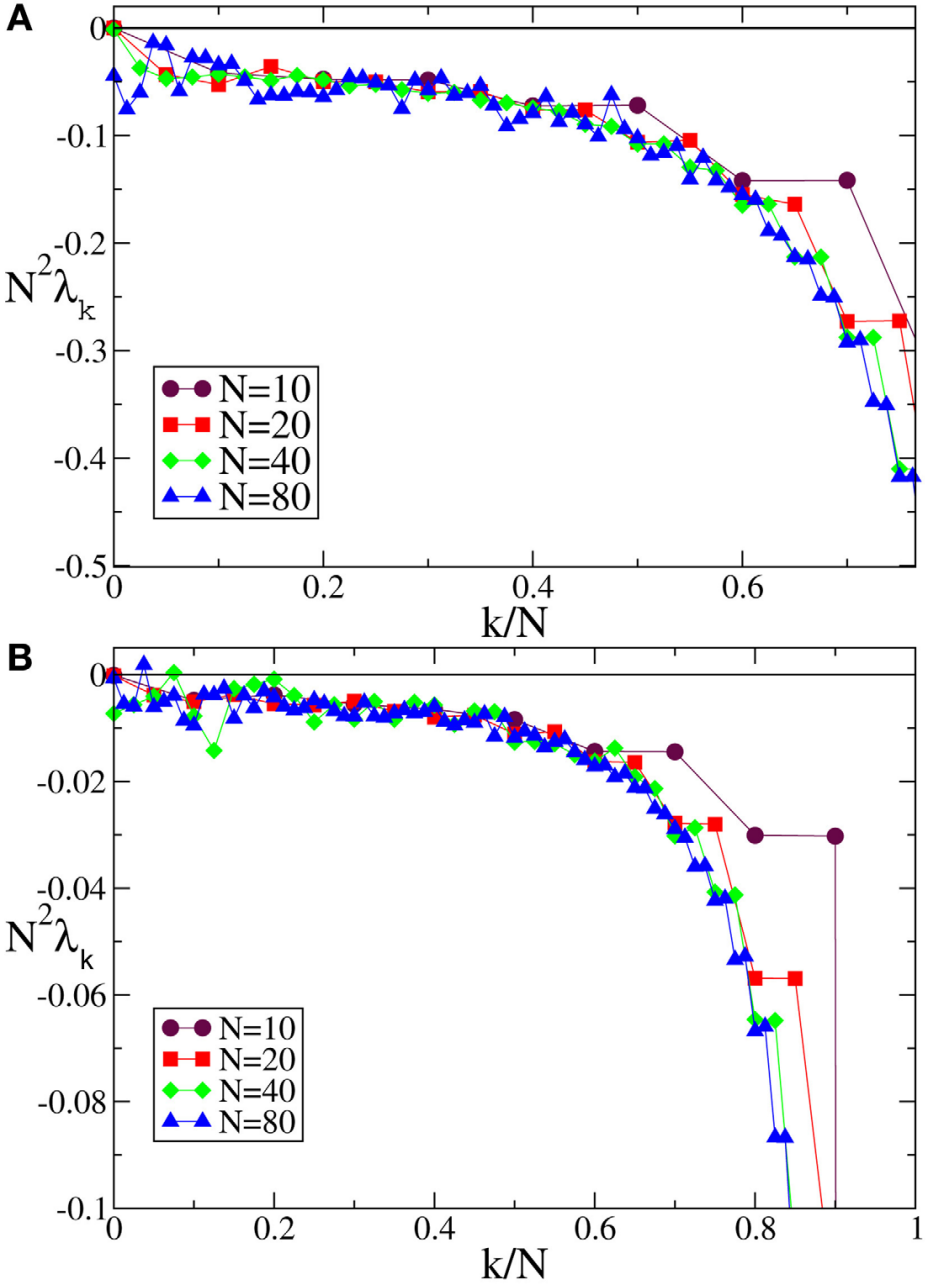
**Lyapunov spectra for neurons forced by an external periodic signal, we observe the scaling 1/*N*^2^ for the discontinuous velocity fields (A) *F*_0_(*X*) and (B) *F*_1_(*X*)**. In both cases *A* = 0.1, *T*_*a*_ = 2.

In the case of *F*_0_ and *F*_1_, we have always found that the Lyapunov spectrum scales as 1/*N*^2^ as theoretically predicted in the absence of external modulation (see Figure 5 for one instance of each of the two velocity fields).

**Figure 6:**
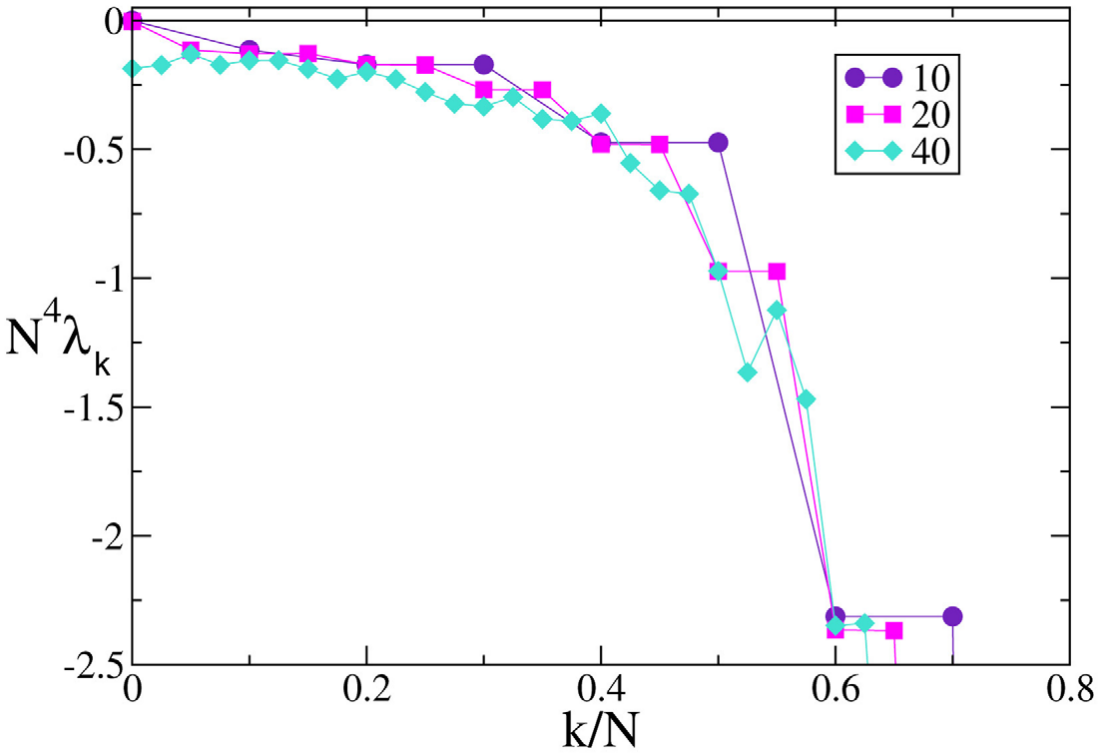
**Lyapunov spectra for neurons forced by an external periodic signal, we observe the scaling 1/*N*^4^ for the continuous velocity field *F*_3_(*X*)**. The data refer to *A* = 0.1, *T*_*a*_ = 2.

A similar agreement is also found for *F*_3_, where the Lyapunov spectrum scales as 1/*N*^4^, exactly as in the absence of external forcing (see Figure 6). Analogous results have been obtained for the other velocity fields (data not shown), which confirm that the validity of the previous analysis extends to more complex dynamical regimes, as long as the membrane potentials are smoothly distributed.

## 7. Summary and open problems

In this paper we have discussed the linear stability of both fully synchronized and splay states in pulse-coupled networks of identical oscillators. By following Abbott and van Vreeswijk (1993), we have obtained analytic expressions for the long-wavelength components of the Floquet spectra of the splay state for generic velocity fields and post synaptic potential profiles. The structure of the spectra depends on the smoothness of both the velocity field and the transmitted pulses. The smoother they are and the faster the eigenvalues decrease with the wavelength of the corresponding eigenvectors. In practice, while splay states arising in LIF neurons with δ-pulses have a finite degree of (in)stability along all directions, those emerging in analytic velocity fields have many exponentially small eigenvalues. These results have been derived in the mean field framework, where the system is assumed to be infinite. Although realistic neural networks are finite, the present analysis predicts correctly, even for finite systems, the stability of the eigenmodes associated to the fastest scales and the order of magnitude of the eigenvalues corresponding to slower time scales. Interestingly, the scaling behavior of the eigenvalues carries over to that of the Lyapunov exponents, when the network is periodically forced, suggesting that our results have a relevance that goes beyond the highly symmetric solutions studied in this paper.

Finally, we derived an analytic expression for the Floquet spectra for the fully synchronous state. In this case the exponents associated to the dynamics of the membrane potentials are all identical, as it happens for the diffusive coupling, but here the result is less trivial, due to the fact that one must take into account that arbitrarily close to the solution, the ordering of the neurons may be different. Moreover, the value of the (degenerate) Floquet exponent coincides with the evaporation exponent (van Vreeswijk, 1996; Pikovsky et al., 2001) whenever the pulses are sufficiently smooth, while for discontinuous pulses (like exponential and δ-spikes) the equivalence is lost (see also di Volo et al., 2013).

For discontinuous velocity fields, another important property that has been confirmed by our analysis is the role of the ratio *R* = *N*/(*T*_0_α) between the width of the single pulse (1/α) and the average interspike interval of the whole network (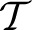 = *T*_0_/*N*). In fact, it turns out that the asynchronous regimes can be strongly stable along all directions only when *R* remains finite in the thermodynamic limit (and is possibly small). This includes the idealized case of δ-like pulses, but also setups where the single pulses are so short that they can be resolved by the single neurons. Mathematically speaking, this result implies that the thermodynamic limit does not commute with the limit of a zero pulse-width. It would be interesting to check to what extent this property extends to more realistic models. A first confirmation result is contained in Pazó and Montbrió (2013), where the authors find a similar property in a network of Winfree oscillators.

Among possible extensions of our analysis, one should definitely mention the inclusion of delay in the pulse transmission. This generalization is far from trivial as it modifies the phase diagram of the possible states (see Bär et al., 2012 for a recent brief overview of the possible scenarios) and it complicates noticeably the stability analysis of the synchronized phase. An analytic treatment of this latter case is reported in Timme et al. (2002) for generic velocity fields and excitatory δ-pulses.

### Conflict of interest statement

The authors declare that the research was conducted in the absence of any commercial or financial relationships that could be construed as a potential conflict of interest.

## Appendices

### A. Fourier components of the phase response curve

In this appendix we briefly outline the way the explicit expression of *A*_*n*_ and *B*_*n*_, defined in Equation (23), can be derived in the large *n* limit for a velocity field *F*(*X*) that is either discontinuous, or continuous with discontinuous first derivatives at the border of the definition interval.

The integration interval [0, 1] appearing in Equation (23) is splitted in *n* sub-intervals of length 1/*n*, and the original equation can be rewritten as

(A1) $$(A_{n}+iB_{n})=\sum_{k=1}^{n}\int_{(k-1)/n}^{k/n}dy\frac{{\text{e}}^{i2\pi ny}}{G(y)}.$$

For *n* sufficiently large we can assume that the variation of 1/*G(y)* is quite limited within each sub-interval, and we can approximate the function as follows, up to the second order

$$\begin{array}{l} \frac{1}{G(y)}=\frac{1}{g+T_{0}F(y_{0})}\{1-\frac{T_{0}F^{\prime }(y_{0})}{g+T_{0}F(y_{0})}(y-y_{0})\\ \;+\left[{\left(\frac{T_{0}F^{\prime }(y_{0})}{g+T_{0}F(y_{0})}\right)}^{2}-\;\frac{T_{0}F^{\prime \prime }(y_{0})}{2(g+T_{0}F(y_{0}))}\right]{\left(y-y_{0}\right)}^{2}\} \end{array}$$

where *y*_0_ = (*k* − 1)/*n* is the lower extremum of the *n*th sub-interval.

By inserting these expansions into Equation (A1) and by performing the integration over the *n* sub-intervals, we can determine an approximate expression for *A*_*n*_ and *B*_*n*_. The estimation of *A*_*n*_ involves integrals containing cos(2π*ny*); it is easy to show that the integral over each sub-interval is zero if the integrand, which multiplies the cosinus term, is constant or linear in *y*; therefore the only non-zero terms are,

(A2) $$\int_{(k-1)/n}^{k/n}dy\cos(2\pi ny)y^{2}=\frac{1}{2\pi^{2}n^{3}}.$$

This allows to rewrite  where

(A4) $$H_{2}(x)=\left[\frac{{\left(T_{0}F^{\prime }(x)\right)}^{2}}{{\left(g+T_{0}F(x)\right)}^{3}}-\frac{T_{0}F^{\prime \prime }(x)}{2(g+T_{0}F{\left(x\right)}^{2})}\right]\text{​}.$$

It is easy to verify that *H*_2_(*x*) admits an exact primitive and therefore to perform the integral appearing in Equation (A3) and to arrive at the expression reported in Equation (28).

The estimation of *B*_*n*_ is more delicate, since now integrals containing sin(2π*ny*) are involved. The only vanishing integrals over the sub-intervals are those with a constant integrand multiplied by the sinus term and therefore the estimation of *B*_*n*_ reduces to

$$\begin{array}{l} B_{n}=\sum_{k=1}^{n}H_{1}\left(\frac{k-1}{n}\right)\int_{(k-1)/n}^{k/n}dy\sin(2\pi ny)y\\ \;+\sum_{k=1}^{n}H_{2}\left(\frac{k-1}{n}\right)\int_{(k-1)/n}^{k/n}dy\sin(2\pi ny)\left(y^{2}-2y\frac{k-1}{n}\right) \end{array}$$

where

(A5) $$H_{1}(x)=-\frac{T_{0}F^{\prime }(x)}{{\left(g+T_{0}F(x)\right)}^{2}},$$

and the non-zero integrals are

(A6) $$\int_{(k-1)/n}^{k/n}dy\sin(2\pi ny)y=-\frac{1}{2\pi n^{2}},$$

and

(A7) $$\int_{(k-1)/n}^{k/n}dy\sin(2\pi ny)y^{2}=\frac{1-2k}{2\pi n^{3}}.$$

This allows to rewrite *B*_*n*_ as

(A8) $$\begin{array}{l} B_{n}=-\frac{1}{2\pi n}\sum_{k=1}^{n}H_{1}\left(\frac{k-1}{n}\right)\frac{1}{n}\\ \;-\frac{1}{2\pi n^{2}}\sum_{k=1}^{n}H_{2}\left(\frac{k-1}{n}\right)\frac{1}{n}. \end{array}$$

We can then return to a continuous variable by rewriting (A8), up to the  (1/*n*^3^), as

(A9) $$\begin{array}{l} B_{n}=-\frac{1}{2\pi n}\left[\int_{0}^{1}H_{1}(x)dx+\frac{H_{1}(1)-H_{1}(0)}{2n}\right]\\ \;-\frac{1}{2\pi n^{2}}\int_{0}^{1}H_{2}(x)dx. \end{array}$$

The expression Equation (29) is finally obtained by noticing that the primitive of *H*_2_(*x*) is *H*_1_(*x*)/2, and that

$$\int_{0}^{1}H_{1}(x)dx=\frac{1}{\left(g+T_{0}F(0)\right)}-\frac{1}{\left(g+T_{0}F(1)\right)}.$$

For continuous velocity fields, *B*_*n*_ = 0 so that, we can derive from Equation (26) an exact expression for the real part of the Floquet spectrum in the case of even *L* (for odd *L* the equivalent expression is given by Equation (31))

(A10) $$Re\{\lambda_{n}\}=\frac{g{\text{KST}}_{0}^{L+1}{\left(-1\right)}^{L/2}}{{\left(2\pi n\right)}^{\left(L+2\right)}}\frac{F^{\prime }(0)-F^{\prime }(1)}{G{\left(1\right)}^{2}}.$$

A rigorous validation of the above formula would require going one order beyond in the 1/*n* expansion of *B*_*n*_, a task that is utterly complicated. In the specific case of the Quadratic Integrate and Fire neuron (or Θ-neuron) *F*(*X*) = *a* − *X*(*X* − 1), it can be, however, analytically verified that *B*_*n*_ is exactly zero. Moreover, Equation (A10) is in very good agreement with the numerically estimated Floquet spectra for two other continuous velocity fields, namely *F*_4_(*X*) and *F*_2_(*X*) as shown in Figures 1, 3, respectively. As a consequence, it is reasonable to conjecture that Equation (29) is correct up to order  (1/*n*^4^).

### B. Evaporation exponent for the LIF model

In this appendix we determine the (left and right) evaporation exponent for a synchronous state of a network of LIF neurons. This is done by estimating how the potential of a probe neuron, forced by the mean field generated by the network activity, converges toward the synchronized state. The stability analysis is performed by following the evolution of a perturbed probe neuron. Let us first consider an initial condition, where the synchronized cluster has just reached the threshold (*X*_*c*_ = 1), while the probe neuron is lagging behind at a distance δ_*i*_. Such a distance is equivalent to a delay *t*_*d*_

(A11) $$t_{d}=\frac{\delta_{i}}{F^{+}(1)},$$

where the subscript “+” means that the velocity field is estimated just after the pulses have been emitted. Over the time *t*_*d*_, the potential of the cluster increases from the reset value 0 to

(A12) $$\delta_{c}=F^{+}(0)t_{d}=\frac{F^{+}(0)}{F^{+}(1)}\delta_{i}.$$

From now on (in LIF neurons), the distance decreases exponentially, reaching the value

(A13) $$\delta_{f}=\delta_{c}e^{-T},$$

after a period *T*. As a result,

(A14) $$\frac{\delta_{f}}{\delta_{i}}=\frac{F^{+}(0)}{F^{+}(1)}e^{-T}=\frac{a+gE^{+}}{a-1+gE^{+}}.$$

The logarithm of the expansion factor gives the left evaporation exponent

(A15) $$\lambda_{e}^{l}=\ln\left(\frac{a+gE^{+}}{a-1+gE^{+}}\right)-T.$$

Let us now consider a probe neuron which precedes the synchronized cluster by an amount δ_*i*_. After a time *T* the distance becomes

(A16) $$\delta_{c}=\delta_{i}e^{-T}$$

since no reset event has meanwhile occurred. Such a distance corresponds to a delay

(A17) $$t_{d}=\frac{\delta_{c}}{F^{-}(1)},$$

where the subscript “−” means that the velocity has now to be estimated just before the pulse emission. By proceeding as before one obtains,

(A18) $$\frac{\delta_{f}}{\delta_{i}}=\frac{F^{-}(0)}{F^{-}(1)}e^{-T}.$$

so that the right evaporation exponent writes

(A19) $$\Lambda_{e}^{r}=\ln\left(\frac{a+gE^{-}}{a-1+gE^{-}}\right)-T.$$

It is easy to see that the left and right exponents differ if and only if *E*^−^ ≠ *E*^+^, i.e., if the pulses themselves are not continuous: this is, for instance, the case of exponential and δ pulses.
